# The Human “Cochlear Battery” – Claudin-11 Barrier and Ion Transport Proteins in the Lateral Wall of the Cochlea

**DOI:** 10.3389/fnmol.2017.00239

**Published:** 2017-08-10

**Authors:** Wei Liu, Annelies Schrott-Fischer, Rudolf Glueckert, Heval Benav, Helge Rask-Andersen

**Affiliations:** ^1^Department of Surgical Sciences, Section of Otolaryngology, Uppsala University Hospital Uppsala, Sweden; ^2^Department of Otolaryngology, Medical University of Innsbruck Innsbruck, Austria; ^3^R&D, MED-EL GmbH Innsbruck, Austria; ^4^Department of Surgical Sciences, Head and Neck Surgery, Section of Otolaryngology, Uppsala University Hospital Uppsala, Sweden

**Keywords:** human, cochlea, stria vascularis, spiral ligament, Claudin-11, structured illumination microscopy

## Abstract

**Background:** The cochlea produces an electric field potential essential for hair cell transduction and hearing. This biological “battery” is situated in the lateral wall of the cochlea and contains molecular machinery that secretes and recycles K^+^ ions. Its functioning depends on junctional proteins that restrict the para-cellular escape of ions. The tight junction protein Claudin-11 has been found to be one of the major constituents of this barrier that maintains ion gradients (Gow et al., 2004; Kitajiri et al., 2004a). We are the first to elucidate the human Claudin-11 framework and the associated ion transport machinery using super-resolution fluorescence illumination microscopy (SR-SIM).

**Methods:** Archival cochleae obtained during meningioma surgery were used for SR-SIM together with transmission electron microscopy after ethical consent.

**Results:** Claudin-11-expressing cells formed parallel tight junction lamellae that insulated the epithelial syncytium of the stria vascularis and extended to the suprastrial region. Intercellular gap junctions were found between the barrier cells and fibrocytes.

**Conclusion:** Transmission electron microscopy, confocal microscopy and SR-SIM revealed exclusive cell specialization in the various subdomains of the lateral wall of the human cochlea. The Claudin-11-expressing cells exhibited both conductor and isolator characteristics, and these micro-porous separators may selectively mediate the movement of charged units to the intrastrial space in a manner that is analogous to a conventional electrochemical “battery.” The function and relevance of this battery for the development of inner ear disease are discussed.

## Introduction

Human hearing depends on approximately 15,000 mechanoreceptors in each cochlea. The functioning of these mechanoreceptors relies on an electric potential generated in the lateral wall (von Bekesy, 1952; Smith et al., 1958; Tasaki and Spyropoulos, 1959; Salt et al., 1987; Zidanic and Brownell, 1990; Dallos et al., 1996; Wangemann, 2002; Mistrík et al., 2009) but also on a high endolymph K^+^ concentration. This field potential, called endocochlear potential (EP), is produced by a multi-layered epithelium called the StV linked to a neighboring fibrocyte network. It houses molecular machinery that secretes K^+^ into the endolymph and recycles these ions.

The StV is surrounded by apical and basal TJ barriers that restrict the para-cellular escape of ions before uptake into the MCs and secretion into the endolymph. The basal barrier permits a transcellular ion flux through GJ channels (Kikuchi et al., 1995, 2000; Takeuchi et al., 2000; Forge et al., 2002, 2003; Liu et al., 2016). The ICs fronting the MCs, express the inwardly rectifying K^+^ channel KCNJ10 (Kir 4.1), playing a pivotal role for EP generation (Hibino et al., 1997). Deletion of the gene causes deafness with diminished K^+^ secretion and loss of the EP (Marcus et al., 2002). Kir4.1 may act as a key regulator that sets the membrane potential via K^+^ diffusion through Ba^2+^-sensitive channels in the ICs (Hibino et al., 1997, 2010; Takeuchi et al., 2000; Nin et al., 2016).

According to Salt et al. (1987) and others (Takeuchi et al., 2000; Nin et al., 2008, 2016), BC TJs are necessary to maintain the EP. This hypothesis was confirmed by *in vivo* experiments showing that Claudin-11 null mice exhibit severe deafness and a low EP but no other morphological changes (Gow et al., 2004; Kitajiri et al., 2004a). Several claudin isoforms exist in the inner ear, but surprisingly, the BC TJs consist exclusively of Claudin-11 (Kitajiri et al., 2004b). Consequently, human hearing may depend on this insulator protein isoform.

We recently presented data on the cellular architecture and ion transport systems in the lateral wall of the human cochlea with special reference to GJ protein genes beta 2 [GJB2/ connexin26 (Cx26)] and 6 [GJB6/connexin30 (Cx30)], commonly involved in hereditary deafness (Liu et al., 2016). In this study, we analyzed the Claudin-11 TJ framework and the associated ion transport machinery in the human cochlea using confocal microscopy, TEM and SR-SIM. SR-SIM offers a volume resolution nearly eightfold higher than that of conventional microscopy (Schermelleh et al., 2008). A combination of ion transport markers was used, such as Na/K-ATPase and NKCC1, which are essential for K^+^ uptake into MCs followed by secretion into the endolymph. The KCNQ1/KCNE1 gene, which encodes the voltage-gated potassium channel Kv7.1 (KvLQT1), is expressed in the apical cell membrane of the MCs (Marcus and Shen, 1994; Wangemann, 1995) and was also verified in the human (Liu et al., 2016).

Our examination revealed Claudin-11-expressing cells exhibiting both conductor and isolator characteristics to selectively mediate the movement of charged units in a manner that is analogous to a conventional electrochemical “battery.” GJs, apart from transferring signaling molecules (Zhang et al., 2005) may act like wires in an electric circuit, ensuring the flow of charged units and the membrane capacitance of the basal surface. Our findings may add to the understanding of human inner ear physiology, EP generation and the possible role of the Claudin-11 barrier in human ear disease.

## Materials and Methods

### The Human Cochlea – A Histological Challenge

A comprehensive analysis of the distribution of the major ion transport proteins in the human cochlea, which are important for recycling and regulating K^+^ homeostasis, was performed by Weber et al. (2001). Their study did not include TJ barrier proteins. The authors used temporal bones collected during autopsy and fixed with 10% buffered formalin solution within 6 h of death. Studies of the human cochlea are demanding because the hard bone necessitates a long period of decalcification. Moreover, well-preserved tissue is difficult to obtain (Glueckert et al., 2005; Rask-Andersen et al., 2012). In the current study, well-fixed human tissue was obtained with excellent antigen retrieval, allowing the study of protein expression using super-resolution microscopy. A disadvantage was the limited amount of tissue and number of sections obtained.

In addition, one pig and one guinea pig cochlea were processed for Claudin-11 staining. Pig and guinea pig cochleae were dissected out, stapes removed and the cochlear perilymph space flushed with fixative before placed in fixative followed by decalcification. The procedure followed that used for human sections including antibodies and staining.

### Ethics Statement

The study of human materials was approved by the local ethics committee (no. 99398, 22/9 1999, cont, 2003, Dnr. 2013/190), and subjects gave informed consent. The study adhered to the rules of the Declaration of Helsinki. Archival sections from adult cochleae were used (Liu et al., 2016). Pig and guinea pig cochleae were also analyzed in parallel. Ethical consent were obtained from the local ethical committee of Uppsala for animal use. The pig study’s protocol was approved (permit number C108/4) by the Regional Animal Review Board of Uppsala, Sweden and guinea pig C98/12 and C66/16.

### Transmission Electron Microscopy

Two archival specimens collected during surgery were analyzed in Innsbruck, Austria. The specimens were fixed in 3% phosphate-buffered glutaraldehyde, pH 7.4, and rinsed in 0.1 M cacodylate buffer, followed by fixing with 1% osmium tetroxide at 4°C for 4 h. The specimens were infiltrated with Epon resin in a vacuum chamber for 4 h. For the TEM analysis, sections were viewed with Zeiss LIBRA (Carl Zeiss: Oberkochen, Germany, Institute of Zoology, Innsbruck) and Philips CM 120 (Division of Anatomy, Histology and Embryology, Innsbruck) transmission electron microscopes.

### Fixation and Sectioning of the Human Cochlea for Immunohistochemistry

Four cochleae (2 from males and 2 from females; ages 44–72 years, **Table 1**) were dissected out as a whole piece instead of being discarded, during petro-clival meningioma surgery (two with normal pure tone thresholds for their age and two with moderate sensorineural hearing loss due to life-threatening tumor compression of the brain stem). The trans-cochlear approach is standard in Uppsala since 1988 (oto-neuro-surgery) in these rare conditions. It has reduced the risk for brain stem complication and with a postero-inferior re-routing of the facial nerve, its severance with post-operative facial nerve paralysis can often be avoided. In addition, the clivus region can be better approached. The petrosectomy was performed by an independent skull base surgeon (AK). The entire surgery is routinely performed as a two-stage procedure. At the first day a total petrosectomy is performed with facial nerve re-routing. The second day the tumor is removed. The entire surgery time is usually around 20 h. Instead of drilling away the cochlea it is dissected out. In the operating room, the cochleae were immediately placed in 4% paraformaldehyde, diluted in 0.1 M phosphate-buffered saline (pH 7.4). After a 24-h fixation, the fixative was replaced with PBS and then with a 0.1 M Na-EDTA solution at pH 7.2 for decalcification. After 4 weeks’ decalcification, the cochleae were rinsed with PBS. The cochleae were placed in 20% sucrose diluted in PBS for overnight for cryo-protection. The cochleae were embedded in Tissue-Tek OCT (Polysciences), rapidly frozen and sectioned at 8–10 μm using a Leica cryostat microtome to obtain frozen sections. The frozen sections were collected on gelatin/chrome alum-coated slides and stored below -70°C before immunohistochemistry (IHC).

**Table 1:** Patient data and functioning of their cochleae.

Age (years)	Gender	PTT	Analysis
44	Female	50 dB (1 kHz to 8 kHz)	IHC
51	Male	Normal	IHC
72	Male	50 dB (2 kHz to 4 kHz)	IHC
67	Female	Normal	IHC
56	Male	Normal	TEM
60	Male	Normal	TEM

*PTT, pure tone threshold*.

### Antibodies and Immunohistochemistry

**Table 2** shows the series of antibodies used in the present study. The immunohistochemistry procedures performed on cochlear sections have been described in previous publications (Liu et al., 2009, 2016). Briefly, the slide-mounted sections were incubated with antibody solution under a humidified atmosphere at 4°C for 20 h. After rinsing with PBS (3 × 5 min), then incubated at room temperature in 0.4% triton X-100 containing 2% bovine serum albumin (BSA) for 40 min. The sections were incubated with secondary antibodies conjugated to Alexa Fluor 488 and 555 (Molecular Probes, Carlsbad, CA, United States) under room temperature, and then counter-stained with the nuclear stain DAPI for 5 min, rinsed with PBS (3 × 5 min) and mounted with Vectashield (Vector Laboratories, Burlingame, CA, United States) mounting medium for confocal microscopy. Half of the sections were mounted with ProLong^®^Gold Antifade Mountant and coverslipped with the specified cover glass (0.17 ± 0.005 mm) required for optically matching the SIM microscope objectives. Primary and secondary antibody controls and labeling controls were used to exclude endogenous labeling or reaction products (Burry, 2011). Control sections were incubated with 2% BSA omitting the primary antibodies. The control experiment revealed no visible staining in any structure of the cochleae. Both wide-field and confocal fluorescence imaging software exhibited sensitive fluorescent saturation indications, thereby avoiding overexposure.

**Table 2:** Antibodies used in the present investigation.

Claudin-11	Polyclonal	1:500	Rabbit	ab53041	Abcam
Occludin	Polyclonal	1:100	Rabbit	NBP1-87402	Novus
ATPase (α1)	Monoclonal	1:50	Mouse	NB300-146	Novus
ATPase (β1)	Monoclonal	1:100	Mouse	Ma3-930	Abcam
ATPase (β2)	Polyclonal	1:50	Rabbit	PA5-26279	Invitrogen
NKCC1	Polyclonal	1:500	Rabbit	ab59791	Abcam
Laminin β2	Monoclonal	1:100	Rat	# 05-206	Millipore
Cx30	Polyclonal	1:100	Rabbit	71-2200	Invitrogen
Cx26	Monoclonal	1:50	Mouse	33-5800	Invitrogen
Cx26	Polyclonal	1:200	Rabbit	ACC-2121	Alomone
KCNJ10	Polyclonal	1:500	Rabbit	APC-035	Alomone

*Anti-Claudin-11, anti-oligodendrocyte-specific protein antibody*.

### Imaging and Photography

The stained sections were investigated with an inverted fluorescence microscope (Nikon TE2000) equipped with a spot digital camera with three filters (for emission spectra maxima at 358, 461, and 555 nm). Image-processing software (NIS Element BR-3.2, Nikon), including image merging and a fluorescence intensity analyzer, was installed on a computer system connected to the microscope, For laser confocal microscopy, we used the same microscope equipped with a three-channel laser emission system (Melles Griot, United States). The optical scanning and image-processing tasks were completed with Nikon EZ-C1 (ver. 3.80) software, including the reconstruction of Z-stack images into projections and 3-D images. SR-SIM was performed with a Zeiss Elyra S.1 SIM system using a 63×/1.4 oil Plan-Apochromat objective (Zeiss), sCMOS camera (PCO Edge) and ZEN 2012 software (Zeiss). Multicolor SR-SIM imaging was achieved with the following laser and filter setup: 1st channel – 405 nm laser excitation and BP 420–480 + LP 750 filter; 2nd channel – 488 nm laser excitation and BP 495–550 + LP750 filter; 3rd channel – 561 nm laser excitation and BP 570–620 + LP 750 filter. To maximize the image quality, five grid rotations and five phases were used for each image plane and channel. The grid size was automatically adjusted by the ZEN software for each wavelength of excitation. SR-SIM images were processed with ZEN software using automatic settings and theoretical point spread function (PSF) calculation. 3-D reconstruction was performed from the SR-SIM dataset with Imaris 8.2 (Bitplane, Zürich, Switzerland). A bright-field channel was merged with fluorescence to visualize the cell borders. The microscope is capable of achieving a lateral (X-Y) resolution of ≈100 nm and an axial (Z) resolution of ≈300 nm (Gustafsson et al., 2008). The resolution of the SIM system in BioVis (Uppsala) was measured with sub-resolution fluorescent beads (40 nm, Zeiss) in the green channel (BP 495–550 + LP750). An average PSF value was obtained from multiple beads with the built-in experiment PSF algorithm of the ZEN software. The typical resolution of the system was 107 nm in the X-Y plane and 394 nm in the Z plane.

## Results

### Human Claudin-11 Barrier

**FIGURE 1:**
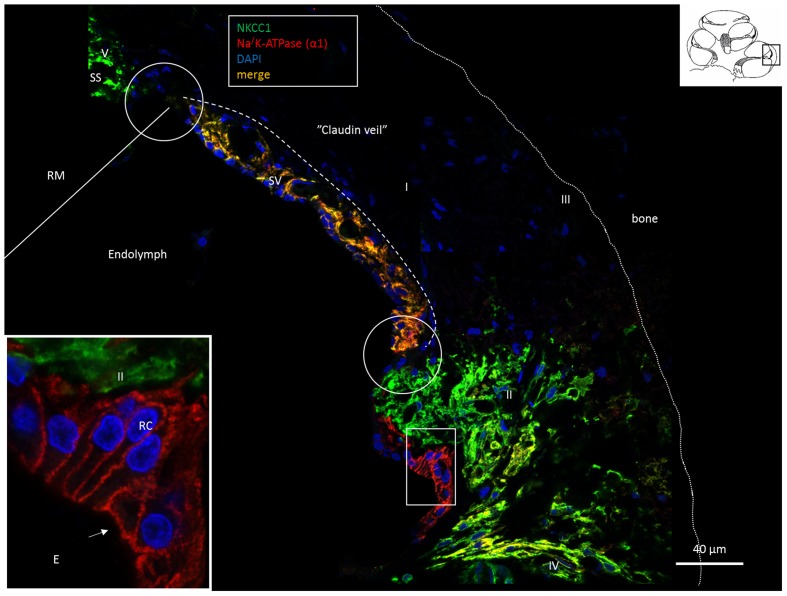
Confocal fluorescence microscopy (overlay) of the lateral wall of the basal turn of the human cochlea. Endolymph secretion by marginal cells is reflected by the dual polarized expression of Na/K-ATPase and the Na/K/Cl cotransporter (merge). Outer sulcus epithelium and root cells (box) express only Na/K-ATPase (inset). Fibrocytes were classified according to Spicer and Schulte (1991) into types I–V after immunohistochemical staining of Na/K-ATPase (α1-subunit) and the Na/K/Cl cotransporter. Type II, IV and V fibrocytes express NKCC1 but also the α1 and β1 Na/K-ATPase isoforms more clearly shown in **Figures 2**, **6**, **8**. Type I fibrocytes lack both enzymes but instead express Cx26/30 through the gap junction network. Gaps in epithelial ion transporter expression are circled. Claudin-11 expressing cells are anchored to the epithelium at these sites, as shown in **Figures 2**, **3**, **7**. Dotted line; location of the Claudin-11 border (“veil”). RC, root cells; SS, suprastrial space; RM, Reissner’s membrane; E, endolymph.

**FIGURE 2:**
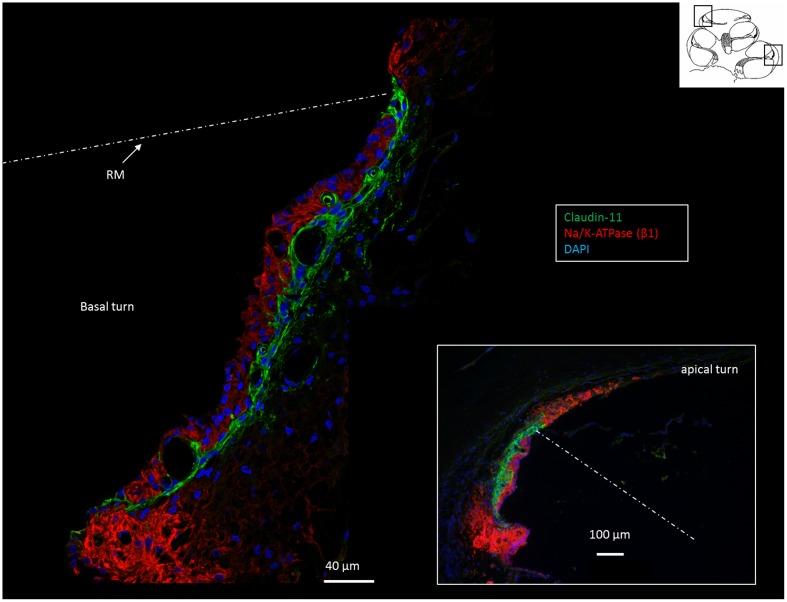
Confocal immunofluorescence of Na/K-ATPase (β1 isoform) and Claudin-11 in the lateral wall of the basal and apical (inset) turns. Claudin-11 is expressed basally from the superior epithelium of the SP to the suprastrial space insulating the K^+^ secreting marginal cell layer. Non-polarized β1 Na/K-ATPase activity is high in type II and type V fibrocytes conceivably reflecting the uptake and recirculation of K^+^. RM, Reissner’s membrane.

**FIGURE 3:**
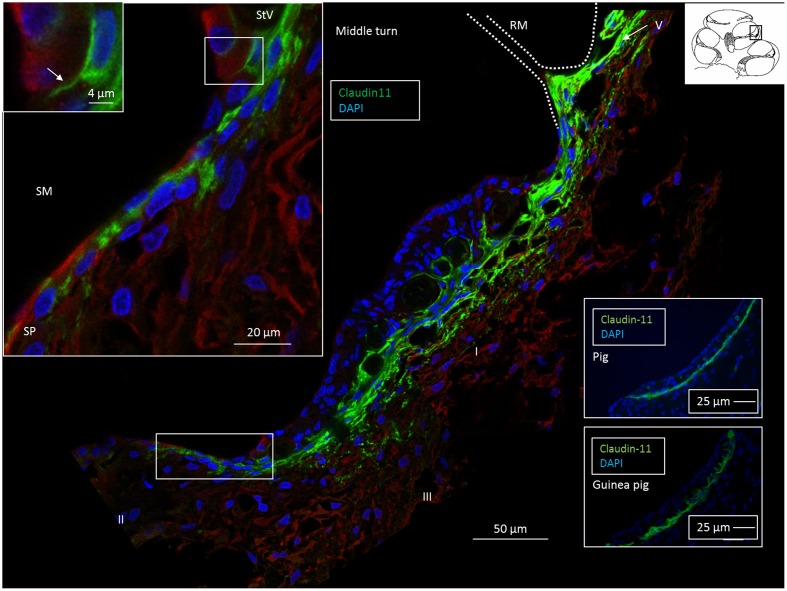
Confocal fluorescence microscopy of the Claudin-11 insulator in the lateral wall of the middle turn of the human cochlea. A band of Claudin-11-expressing basal cells is observed against the StV. The protein is also localized in cells at and superior to Reissner’s membrane (RM). Positive cells extend to the superior epithelium of the SP (boxed area). Left inset: Claudin-11 is expressed between the SP epithelial cells (boxed area). Framed area shows Claudin-11 cell squeezed between SP epithelial cells and is magnified at top left. SM, scala media. Right insets: Claudin-11 staining of StV basal cells in a pig and guinea pig. StV, stria vascularis; SM, scala media; SP, spiral prominence.

**FIGURE 4:**
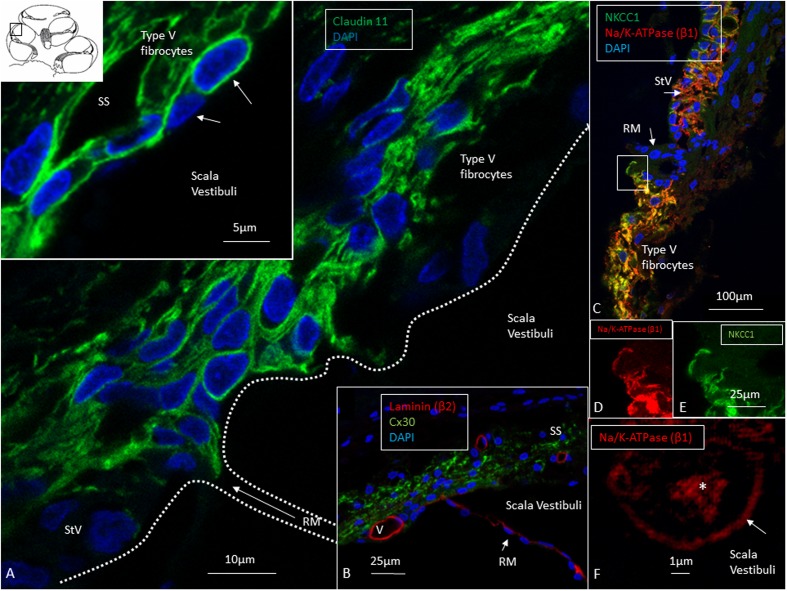
Confocal and super-resolution (SIM) microscopy of the suprastrial space (SS). **(A)** Insulating type V fibrocytes express Claudin-11. The left inset shows cells facing the scala vestibuli (arrows). **(B)** Cx30 expression also reaches the SS reflecting conceivable transcellular recirculation of K^+^ from SV back to the StV. Laminin β2 stains the basal lamina of the RM and capillaries. **(C)** Polarized expression of NKCC1 and Na/K-ATPase (β1) in the StV and non-polarized manifestation in the suprastrial space may reflect the alternate directions of ion flow. Framed area is magnified in **(D,E)** and show expression of the NKCC1 and Na/K-ATPase (β1) membrane transporters facing SV. **(F)** The type V fibrocyte facing the SV shows both intracellular (^∗^) and membrane expression (arrow) of Na/K-ATPase (SR-SIM). StV, stria vascularis; RM, Reissner’s membrane; V, stria vessel; SV, scala vestibuli.

**FIGURE 5:**
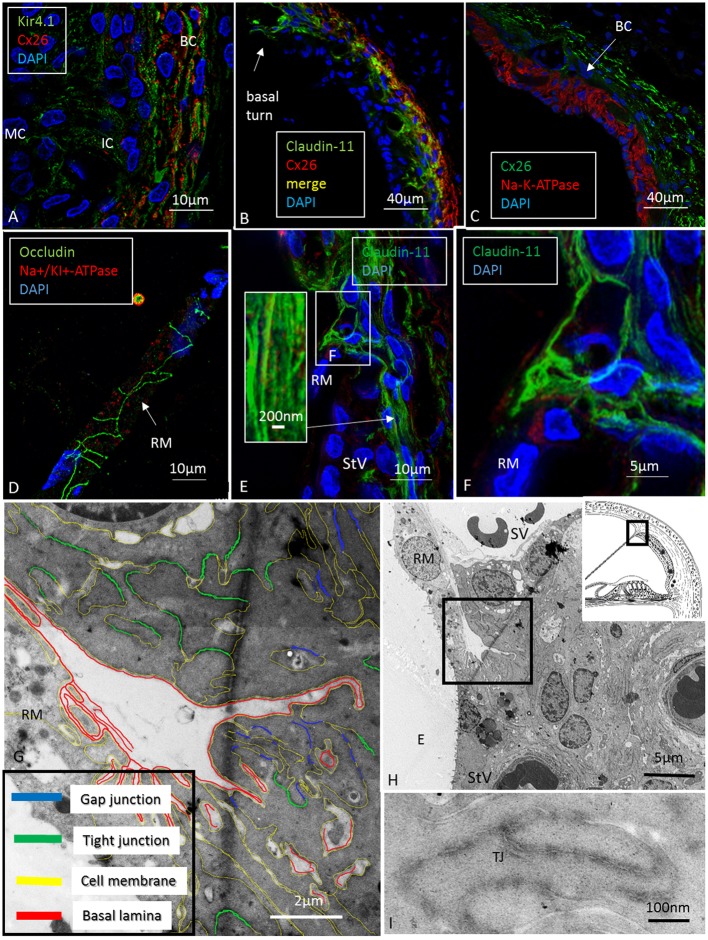
Confocal, super-resolution (SR-SIM) and transmission electron microscopy of the human stria vascularis. **(A)** Co-staining of Kir4.1 and Cx26 in the human StV after SR-SIM processing. The expression pattern may suggest that K^+^ conductive property in the human stria also depends on K^+^ channels located in the basal cells. BC, basal cells; IC, intermediate cells. **(B)** Claudin-11 and Cx26 are co-expressed consistent with TEM findings. **(C)** Cx26 and Na/K-ATPase are separately expressed with no expression of Na/K-ATPase in the syncytial layer. **(D)** Tight junction protein occludin is expressed between epithelial cells in RM (SR-SIM). There is no Claudin-11 expression. **(E)** Instead, Claudin-11 expressing cells bulge into the RM/StV junction for insulation (SR-SIM). The inset shows parallel Claudin-11-positive strands. **(F)** The boxed area in E viewed at higher magnification. **(G,H)** TEM showing the conductor/isolator characteristics of StV epithelial cells with concurrent TJs and GJs. **(I)** Higher magnification of TJs with several membrane fusions between adjacent cells. RM, Reissner’s membrane; SV, scala vestibuli; StV, stria vascularis; E, endolymph; TJ, tight junction.

Confocal microscopy was used to detect pumps, ion transporters and channels in the various domains and helped to define the Claudin-11 barrier in the lateral wall. The basolateral cell membranes of MCs expressed both the α1 and β1 isoforms of Na/K-ATPase and NKCC1, while the OS cells expressed only Na/K-ATPase (**Figures 1**, **2**). The α1 and β1 isoforms were also detected in type II, IV and V fibrocytes. Type I and III fibrocytes were negative for these isoforms. There were breaches in the epithelial expression of this protein at the superior epithelium of the SP and in almost all cases at the lateral insertion of RM. At these gaps, the epithelium expressed Claudin-11 (**Figures 2**, **3**). Claudin-11 was expressed by the BCs of the StV, forming an irregular wall against the lateral surface of the StV (**Figures 2**, **3**). This wall extended to the suprastrial region, where Na/K-ATPase, NKCC1 and Cx26/30 were also expressed (**Figures 2**–**4**, **5B** and **Supplementary Figure S1**). Claudin-11 staining also stretched to the superior epithelium of the SP, where it formed a curvilinear attachment socket (**Figure 3**). Claudin-11 layer usually faced the lateral circumference of the stria vessels but sometimes completely enclosed them, especially those situated more lateral (**Figures 2**, **3**). In the lateral wall, Cx26 was localized with Claudin-11 but was also localized separately in type I fibrocytes (**Figures 5B,C**). Wide-field fluorescence structured-illumination microscopy showed that the Claudin-11 TJ framework consists of multiple parallel strands (**Figures 4**, **5E,F**). Thin branches of Claudin-11 cells could also be found deeper in the MC layer. There was no expression of occludin within the Claudin-11 layer. The association with the ICs could not be convincingly established. The potassium channel Kir4.1 was localized in the intermediate but also the BCs (**Figure 5A**). Occludin, but not Claudin-11, was localized in apical epithelial TJs (**Figure 8C**). Occludin was expressed between the endothelial cells of the StV vessels.

**FIGURE 6:**
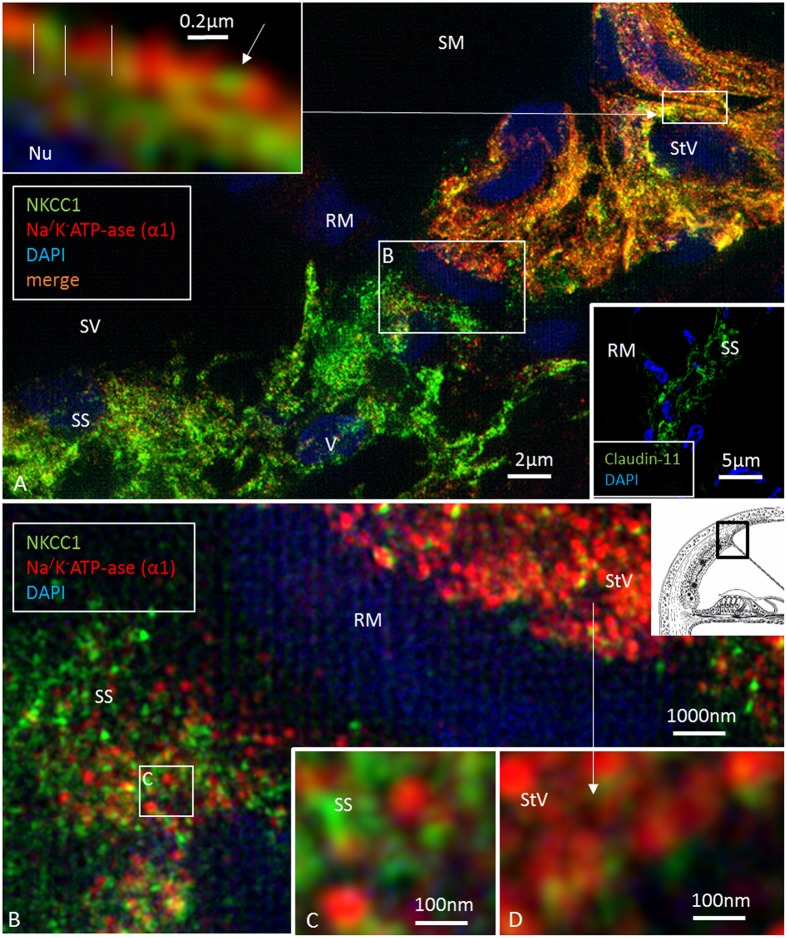
Super-resolution structured illumination microscopy of the RM/StV junction at the basal turn (single optical sections). **(A)** Na/K-ATPase (α1-subunit) and the Na/K/Cl cotransporter are highly expressed in the basolateral cell membrane of the marginal cells. The area in the upper box is magnified in the inset (top left). Na/K/Cl labeling appears together with Na/K-ATPase labeling in an alternating fashion. There is also some sub-membranous staining. Type V fibrocytes of the suprastrial space (SS) show high expression of the Na/K/Cl cotransporter and some Na/K-ATPase. Lower inset shows corresponding expression of Claudin-11 insulating the RM area. The area in the lower box is shown at a higher magnification in **(B)**. **(B)** Large numbers of aggregates of Na/K-ATPase and Na/K/Cl protein are seen in the StV (shown at a higher magnification in **D**). **(C)** The boxed area in **(B)** is shown at a higher magnification. The type V fibrocytes in the SS contains aggregates of non-polarized Na/K-ATPase associated with the Na/K/Cl cotransporter.

At the RM, strands projected a short distance between the epithelial and mesothelial layers (**Figure 5F**). TJs, otherwise, expressed occludin but not Claudin-11 (**Figure 5D**). The Claudin-11 cells were lined by a basal lamina (**Figures 5G–I**) and displayed both GJs and TJs. TEM showed labyrinths of intercellular clefts where membranes were richly decorated with both TJs and GJs (**Figures 5G–I**). TJ complexes consisted of several focal adhesions between outer cell membranes with subjacent electron densities (**Figure 5I**). Suprastrial cells strongly expressed NKCC1 in a non-polarized fashion, together with Na/K-ATPase (**Figure 4C**). In some instances, Na/K-ATPase and NKCC1 expression abutted the StV/RM junction (**Figure 6**). Polyclonal antisera against the Na/K-ATPase α1 subunit and NKCC1 revealed a crowded “pearl-string” of alternating protein aggregates that outlined the entire basolateral membranes of the MCs, exposing some sub-membrane aggregates (**Figure 6A**, top inset). At low magnification and maximum intensity projection, the proteins appeared to be co-labeled; however, at high magnification and in single optical sections, they were separate and formed joined clusters. The aggregates were approximately 100–200 nm.

**FIGURE 7:**
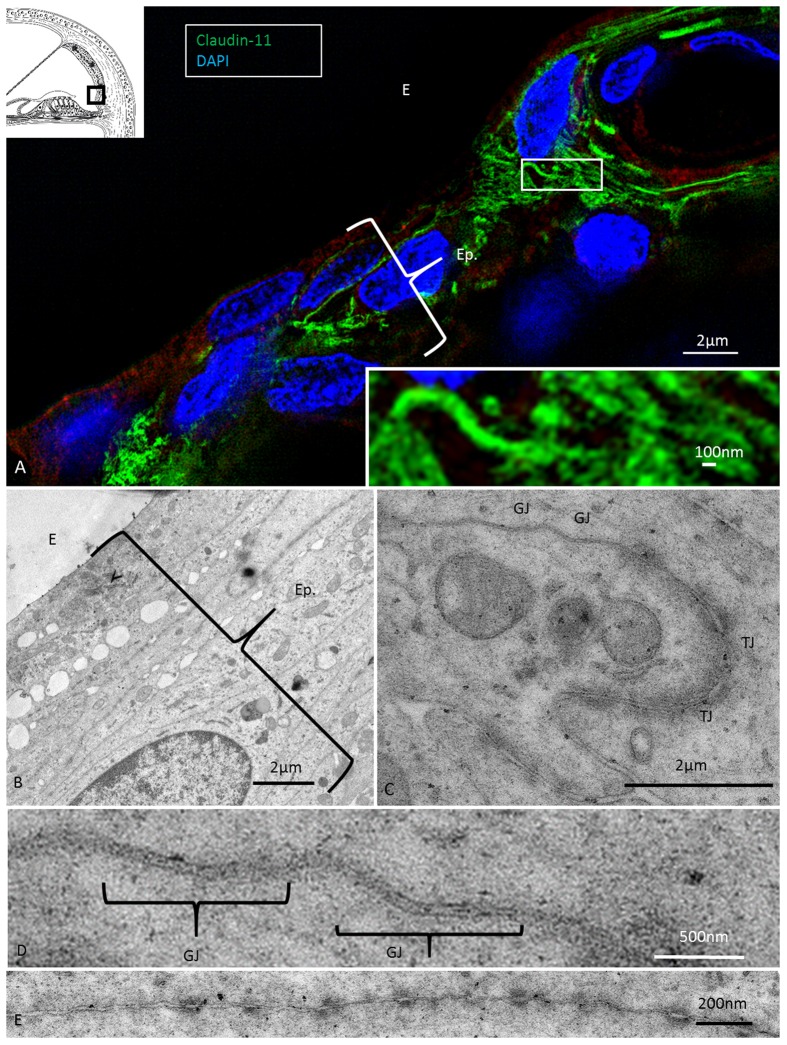
Super-resolution (SR-SIM) and transmission electron microscopy of the infrastrial Claudin-11 insulator. **(A)** Claudin-11 seals the upper epithelium of the SP. Framed area is shown at higher magnification in inset. TJs consist of parallel strands expressing Claudin-11. **(B)** TEM of the corresponding area show tightly arranged epithelial cells decorated with multiple GJs and serially arranged TJs **(C,D)** and desmosome-like intercellular adhesions **(E)**.

**FIGURE 8:**
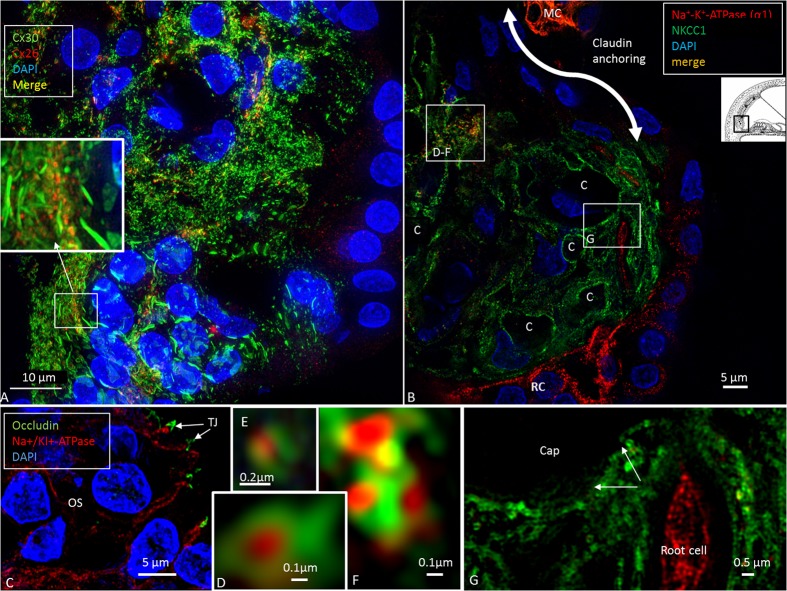
The infrastrial Claudin-11 insulator and Potassium Recirculation. SR-SIM (single optical section) of the spiral prominence (SP) of the basal turn. Channel, ion transporter and barrier distribution highlighting this area for trans-epithelial K^+^ flux and recycling. **(A)** There are two morphologically different Cx30 systems in the outer sulcus epithelium. The root cell bodies show large plaques, while their processes (inset) have smaller plaques that are co-expressed with Cx26. Type II fibrocytes also have smaller GJ plaques associated with Cx26. **(B)** SR-SIM (single optical section) of the SP with co-labeling for Na/K-ATPase (α1-subunit) and the Na/K/Cl cotransporter. High magnification of the protein aggregates **(D–F)**. Co-expression of the marginal cells of the StV can be seen at the top (MC). The lower portion of the SP epithelium expresses only Na/K-ATPase. Apical tight junctions express occludin **(C)**. The root cell processes are rich in Na/K-ATPase and reach the capillary area **(G)**. Type II fibrocytes are rich in Na/K/Cl, particularly around the blood vessels.

At the superior epithelium of the SP, cells with a multitude of parallel Claudin-11 strands surrounded the inferior pole of the StV and were integrated with the epithelium (**Figure 7**). Electron microscopy showed tightly arranged folded cells with an elaborate system of not only TJ strands but also several GJs (**Figures 7B–E**). Desmosome-like structures were also seen (**Figure 7E**). The TJs consisted of multiple membrane fusions with sub-plasma densities (**Figure 7C**). The Claudin-11 cells separated type II fibrocytes from the StV. No Claudin-11 expression was seen between type I and II fibrocytes. Instead, these fibrocytes formed a communicating Cx30 GJ network (**Supplementary Figure S1**). Type II fibrocytes expressed NKCC1 and the α1 and β1 isoforms of Na/K-ATPase (**Figures 8B,D–F**). NKCC1 staining was non-polarized with associated NKCC1 and Na/K-ATPase aggregates of various shapes. The NKCC1 staining was particularly robust at the SP vessels (**Figure 8B** and **Supplementary Figure S2**). The basolateral cell membranes of the OS epithelium contained Na/K-ATPase with a few NKCC1 aggregates. The RCs showed intracellular Na/K-ATPase staining and no NKCC1 (**Figure 8F**). Cx30 was highly expressed in type II fibrocytes as well as between OS cells and root processes (**Figure 8A**). Cx26 was expressed but to a less degree in the type II fibrocytes.

### Pig and Guinea Pig

A well-defined band of Claudin-11 positive cells was seen in the StV in the pig and guinea pig. The stained layers were thinner and seemed to be restricted to a defined layer of BCs. There was no expression in the suprastrial regions (**Figure 3**, insets). The endosteal layer of the SV, medial to the insertion of the RM, expressed Claudin-11 in the pig. Na/K-ATPase β1 was similarly expressed in the MCs of the StV and in type II, IV and V fibrocytes but not in type I and III fibrocytes (**Supplementary Figure S3**).

## Discussion

### Claudin-11 – An Electric Isolator and Conductor

**FIGURE 9:**
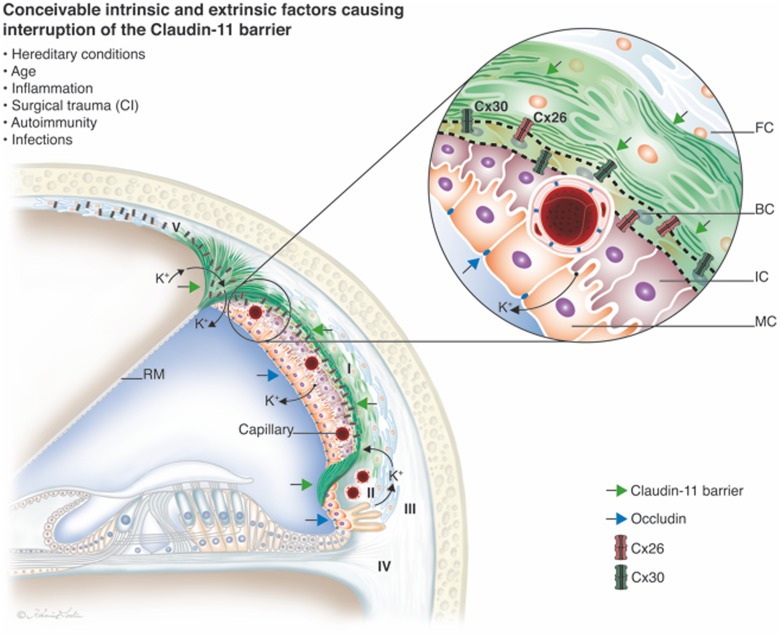
Claudin-11 barrier in the lateral wall of the human cochlea. The protein forms a tight boundary around the StV. It extends from the suprastrial region to the superior epithelium of the SP. Occludin is present in apical tight junctions and between endothelial cells.

The results show the remarkable diversity in the subdomains of the human cochlea lateral wall, as is shown by immunolabeling. This finding supports earlier data regarding specialized fibrocyte subpopulations involved in K^+^ recycling and EP generation (Spicer and Schulte, 1991, 1996; Minowa et al., 1999; Steel, 1999; Weber et al., 2001). The classification of human fibrocytes was inferred from the classification in animals (Spicer and Schulte, 1996) even though the immune expression in humans is not equal to that in rodent models. As previously reported, variations occur both among species and among the different turns of the cochlea. Their immune staining assisted in the delineation and definition of the Claudin-11 layer. This TJ protein also known as oligodendrocyte-specific protein (OSP, Morita et al., 1999; Krause et al., 2008), was expressed in a condensed lamellar layer of BCs but, surprisingly, also in adjacent fibrocytes and supra- and infrastrial fibrocytes (**Figure 9**). A similar expression could not be seen in the pig and guinea pig. The barrier, consisting of BCs and type I fibrocytes, seemed even more prominent in humans compared to animals analyzed in the present study. Contrary to Gow et al. (2004) and Kitajiri et al. (2004a,b), we found no co-expression of occludin and Claudin-11, suggesting that the barrier is even more dependent on Claudin-11 in man. Type I fibrocytes and BCs contained large numbers of GJ complexes expressing Cx26 and Cx30 separately in accordance with earlier findings (Liu et al., 2016). GJs may allow ions and small substances such as cAMP, nucleotides and inositol triphosphate to be transferred across the wall of Claudin-11-expressing cells.

Epithelia contain several Claudin isoforms, but only Claudin-11 is expressed in the BCs of the ear (Gow et al., 2004; Kitajiri et al., 2004b). The role of Claudin-11 in the generation/maintenance of the EP, but not in the high K^+^ concentration in the endolymph, was demonstrated by Gow et al. (2004) and Kitajiri et al. (2004b). This specificity is explained by the ‘two-cell model’ suggested by Salt et al. (1987) and the updated five-compartment (two-cell) model, where the StV is separated by four membranes for the generation of the EP (Takeuchi et al., 2000; Nin et al., 2008, 2016). The BCs form a TJ barrier between the intrastrial space and the electrical syncytium (defined as type I fibrocytes, basal and ICs and endothelial cells), connected with GJs, and thereby plays a crucial role for EP generation and isolation, avoiding short-circuit to perilymph.

*In vivo* analyses demonstrate that EP may stem primarily from two K^+^ diffusion potentials situated at the apical membrane of the MCs and the medial surface of the ICs (Kir4.1). K^+^ seems to be transported from the intrastrial space into the MCs via basolateral Na/K-ATPase and NKCC1 and then secreted into the endolymph via apical KCNQ1/KCNE1 channels (Wangemann et al., 1995; Shen et al., 1997; Liu et al., 2016). According to Adachi et al. (2013) Na/K-ATPases occur at the basolateral surface of the fibrocytes that can maintain high [K^+^] in the syncytial layer by K^+^ uptake to maintain the EP. The unidirectional K^+^ transport to the endolymph is thought to depend on both NKCCs and Na/K-ATPase on the basolateral surfaces of the syncytial and apical MC layers. Recently, however, evidence was provided that the syncytial NKCC1 may be “silent” under physiological conditions (Yoshida et al., 2015). Cochlear fibrocytes have a high positive resting membrane potential explained by a unique Na^+^ permeability (Yoshida et al., 2016). It is thought to be critical for the Kir4.1-induced K^+^ diffusion potential on the IC membranes to produce the EP. The channels or transporters behind this Na^+^ permeability, however, are not known. We found that human type I fibrocytes did not express NKCC1 or any of the Na/K-ATPase isoforms as postulated by several authors (Nin et al., 2008, 2016; Yoshida et al., 2015). Based on our findings we hypothesize that Na/K-ATPase and NKCC1 in type II, IV and V fibrocytes absorb extra-cellular K^+^ and link them into type I fibrocytes and the syncytium via the extensive GJ network (**Supplementary Figure S1**). Type I fibrocytes thereby play a central role in relaying ions back into the StV through a network of GJs, including type II, IV and V fibrocytes.

### Suprastrial Barrier and K^+^ Recycling from Perilymph

The Claudin-11-positive cells were found to project into the RM and the suprastrial space. Schulte and Adams (1989) and others described Na/K-ATPase-positive fibrocytes in the suprastrial region, and these cells were thought to play a key role in the uptake and concentration of K^+^ from the SV perilymph and its intracellular return to type I fibrocytes and the StV. We found a similar arrangement in humans, with non-polar NKCC1 expression associated with high Na/K-ATPase activity (α1β1) and Cx26/30-expressing GJs. There was also a layer of cells that did not express Claudin-11 but bordered the perilymph. This arrangement may suggest K^+^/Na^+^ exchange and then K^+^ uptake from these cells, which is relayed into an intracellular GJ network back to the StV. The Claudin-11 barrier may secure the tightness of the extracellular space and protect against ion contamination of the spiral ligament (**Figure 9**).

### Infrastrial Barrier and Lateral K^+^ Recycling

We found Claudin-11 cells anchored to the SP, separating it from the StV (**Figures 2**, **7**, **9**). The barrier may isolate sequestered K^+^ and support the notion of a K^+^ recirculation from the hair cell region to the SP via multiple GJs between supporting cells (Liu et al., 2017). This is further strengthened by the large number of GJs between subpopulations of fibrocytes forming a connecting network to the StV (**Supplementary Figure S1**; Liu et al., 2016). StV K^+^ is thought to be derived mainly from the perilymph through recirculation (Konishi et al., 1978; Salt and Konishi, 1986), but the mode and driving force of this are a matter of debate. Lateral K^+^ recycling from outer hair cells is assumed to be mediated by electrochemical gradients along GJs to the OS and from RCs into the spiral ligament, where K^+^ is taken up by type II fibrocytes (Hibino and Kurachi, 2006; Jagger et al., 2010). From here, K^+^ may be relayed via the GJ system in type I fibrocytes to the StV through electrochemical gradients (Schulte and Adams, 1989; Kikuchi et al., 1995; Crouch et al., 1997; Sakaguchi et al., 1998). A passive transfer of K^+^ to the StV is less likely because there is no gradient (Salt et al., 1987; Ikeda and Morizono, 1989). BK channels were found in type I fibrocytes (Shen et al., 2004), and these may be responsible for transporting K^+^ back to the StV through their large K^+^ membrane conductance (Liang et al., 2003). Transfer across RCs may be mediated by Kir4.1 channels (Jagger et al., 2010; Eckhard et al., 2012), whose resting conductance is dominated by K^+^ channels. We could not successfully verify Kir4.1 in these cells in the present investigation. The polarized expression of Na/K-ATPase in the basolateral membrane of the OS epithelial cells with little NKCC1 expression contrasted to their joint appearance in the MCs. The functional significance of this findings remains to be elucidated. In the OS epithelium, we found two distinct Cx30 GJ systems (**Figure 8**). One was between RC bodies, with 2–5 micron diameter plaques, and the other was between RC processes with smaller plaques associated with Cx26 GJs (**Figure 8A**). Type II fibrocyte GJs resembled those in root processes but were separated by a basal lamina. This pattern was recently described in the human cochlea (Liu et al., 2017).

Type II fibrocytes expressed the secretory isoform of NKCC1, as shown previously (Weber et al., 2001) and in animal models (Crouch et al., 1997; Delpire et al., 1999), together with α1β1 Na/K-ATPase. SR-SIM demonstrated an association between these aggregates suggesting tissue-specific ion regulation. Na/K-ATPase uses Na^+^ provided by NKCC1 for K^+^ uptake. According to ten Cate et al. (1994), the α1β1 subunit combination of Na/K-ATPase is more common in the spiral ligament, while α1β2 is prevalent in the StV. Fibrocytes in the SP may express either the α1 or α2-isoform or both with some transition during development (McGuirt and Schulte, 1994; Peters et al., 2001). In the gerbil and mouse, the α2 subunit was identified in subpopulations of type II fibrocytes lying immediately beneath the SP. It was speculated that the conditions are similar to the high concentrations of extracellular K^+^ in the interstitial fluid around astrocytes in the brain and play an important role in the uptake of K^+^ for intracellular transport to the StV (McGuirt and Schulte, 1994; Schulte and Steel, 1994). We found high expression of β1, suggesting that type II fibrocytes exhibit the α1β1 combination. In animal models (Kikuchi et al., 1995; Crouch et al., 1997) and in humans (Weber et al., 2001), there was high expression of Na/K-ATPase in type II fibrocytes and moderate-to-strong expression of NKCC1. Notably, in the present study, NKCC1 was strongly expressed in and around the SP capillaries, suggesting that K^+^ ions may also escape into these vessels, which may act as a safety valve for excessive loads of K^+^ ions sequestered in the SP under various conditions (**Supplementary Figure S2**). The prominence of the SP could be explained by turgor variations. According to Spicer and Schulte (1996), subpopulations of type II fibrocytes commonly border blood vessels in the SP, suggesting that these fibrocytes may contribute to transport between the StV and blood.

### Type IV Fibrocytes

Consistent with earlier reports (Schulte and Adams, 1989), Na/K-ATPase was also expressed in type IV fibrocytes facing the ST. Here, we found no Claudin-11, and the reason for this discrepancy may be the small K^+^ gradient existing across this border. According to Sterkers et al. (1988), the K^+^ concentration in the ST perilymph is lower (3.4 mM) than in the SV (6–8 mM). This difference makes a Claudin-11 barrier along the type IV fibrocytes unnecessary. Furthermore, these cells were segregated and had no intercellular GJs.

### The “Cochlear Battery” and Claudin-11 – Role in Ear Disease

A mounting number of diseases are being associated with alterations in intercellular TJs, such as autoimmunity caused by unsolicited trans-epithelial antigen passage in the gut, cancer development, infections and allergies (Cereijido et al., 2007). Epithelial apical TJs play an important role to maintain the high ion concentration gradients between the endolymph and perilymph (Jahnke, 1975; Riazuddin et al., 2006). The StV is a unique type of epithelium containing cells of various derivations and functions. The origin of the BCs is uncertain, and they are not lined by a continuous basal lamina and therefore have no clear separation from the underlying connective tissue. According to Trowe et al. (2011), who employed genetic lineage tracing, these BCs originate from spiral ligament fibrocytes. This origin may explain the unusual expression pattern of Claudin-11. The Cldn11 gene, which encodes Claudin-11, has not, thus far, been identified as a deafness gene while mutations in the genes encoding Claudin-14 (DFNB29) and Claudin-9 cause autosomal recessive deafness in humans and mice (Wilcox et al., 2001; Nakano et al., 2009). Both claudins are expressed in the apical TJs of the sensory epithelium of the human organ of Corti.

The intrastrial space is separated by an unbroken fortification of TJ protein, and its lack of redundancy may suggest that cochlear function rely on Claudin-11, thereby playing an important role in human inner ear disease. Most epithelia in the inner ear co-express 6–9 claudin isoforms, but only Claudin-11 is detectable in the BCs (Kitajiri et al., 2004a). A breach of the para-cellular barrier (temporary or permanent) in any region opens the intrastrial space and eradicates the electrical isolation, leading to reduced EP and hearing loss (**Figure 9**). In a model for the electrochemistry and K^+^ circulation of charged units (K^+^) in the human cochlear lateral wall Claudin-11 TJ protein may form a filter and isolator to avoid ion reflux. Both intrinsic and extrinsic factors may be responsible for its interruption, such as hereditary conditions, inflammation, trauma, autoimmunity and infections. Claudin-11 is expressed in TJs in myelin sheaths in the brain and Sertoli cells in the testis (Gow et al., 1999; Morita et al., 1999). There is evidence that Claudin-11 can act as an autoantigen in the development of autoimmune demyelinating disease (Bronstein et al., 2000). The new era of inner ear surgery in combination with hearing preservation and novel atraumatic principles also highlights the importance of understanding this system for achieving optimal results.

## Conclusion

The lateral wall of the cochlea contains a molecular machinery that secretes and recycles K^+^ ions. The cell system is essential for generation of the exceptional EP and hair cell transduction. We describe the Claudin-11 molecular framework that insulates the ion transport machinery in the human. It may prevent leakage but equally exhibits conductor characteristics, allowing recycling of K^+^ through ion-permissive GJs for conceivable incessant “recharge.” Interruption of this “battery insulator” may cause sensorineural deafness. More information on the biology of the Claudin-11 layer and its involvement in ear disease are warranted.

## Author Contributions

WL provided most of the experiments’ results, participated in composition of the manuscript. AS-F and RG provided TEM facilities and performed ultrastructural electron microscopic analyses. RG performed TEM and provided photos. HB provided ideas for the research. HR-A supervising the research, organized the manuscript and the main writer and performed transmission electron microscopic analyses.

## Conflict of Interest Statement

HB is affiliated with a commercial company, MED-EL GmbH. The study was also partly funded by MED-EL GmbH. The other authors declare that the research was conducted in the absence of any commercial or financial relationships that could be construed as a potential conflict of interest.

## Supplementary Material

FIGURE S1 Cx30 expressing GJs form a connecting syncytium between subpopulations of fibrocytes (I, II and V) to the StV. Epithelial Cx30 expression is also seen in supporting cells of the organ of Corti, Claudius cells and outer sulcus cells. The Claudin-11 barrier is outlined (dotted line) with its anchoring sites (bold arrows).

FIGURE S2 Combined bright-field and SR-SIM shows Na/K/Cl cotransporter expressed around the SP blood vessels. The same region shown as in **Figure 8B** (the inset shows the boxed area at a higher magnification). The root cell process (RP) expresses Na/K-ATPase (α1-subunit). SP, spiral prominence; V, vessels in the spiral prominence.

FIGURE S3 Na/K-ATPase (β1-subunit) and Claudin-11 expression in the pig cochlea. **(A)** Na/K-ATPase is heavily expressed in the type II and V fibrocytes and in the stria vascularis marginal cells. There is also a strong expression in the organ of Corti and spiral limbus (displaced tympanic covering layer). **(B)** Claudin is expressed in a thin band reaching from the spiral prominence to the region near the Reissner’s membrane. There is no Claudin-11 expression in the suprastrial region (^∗^; StV epithelium slightly separated). **(C)** Confocal microscopy. There is no Claudin expression at a region (gap) beneath the insertion point of RM (arrow). SP, spiral prominence; StV, stria vascularis. Type I, II and V fibrocytes. RM, Reissner’s membrane.

## Abbreviations

BC: Basal cell
BM: Basilar membrane
DAPI 4′: 6-Diamidino-2-phenylindole dihydrochloride
E: Endolymph
EDTA: Ethylene-diamine-tetra-acetic acid
GJ: Gap junction
IC: Intermediate cell
MC: Marginal cell
OS: Outer sulcus
RC: Root cell
RM: Reissner’s membrane
SM: Scala media
SP: Spiral prominence
SR-SIM: Super-resolution structured illumination fluorescence microscopy
ST: Scala tympani
StV: Stria vascularis
SV: Scala vestibuli
TEM: Transmission electron microscopy
TJ: Tight junction.
