# Supplementary figures and images for: The Human “Cochlear Battery” – Claudin-11 Barrier and Ion Transport Proteins in the Lateral Wall of the Cochlea
Source: Front Mol Neurosci. 2017 Aug 10;10:239. doi: 10.3389/fnmol.2017.00239 (PMC5554435; doi:10.3389/fnmol.2017.00239)

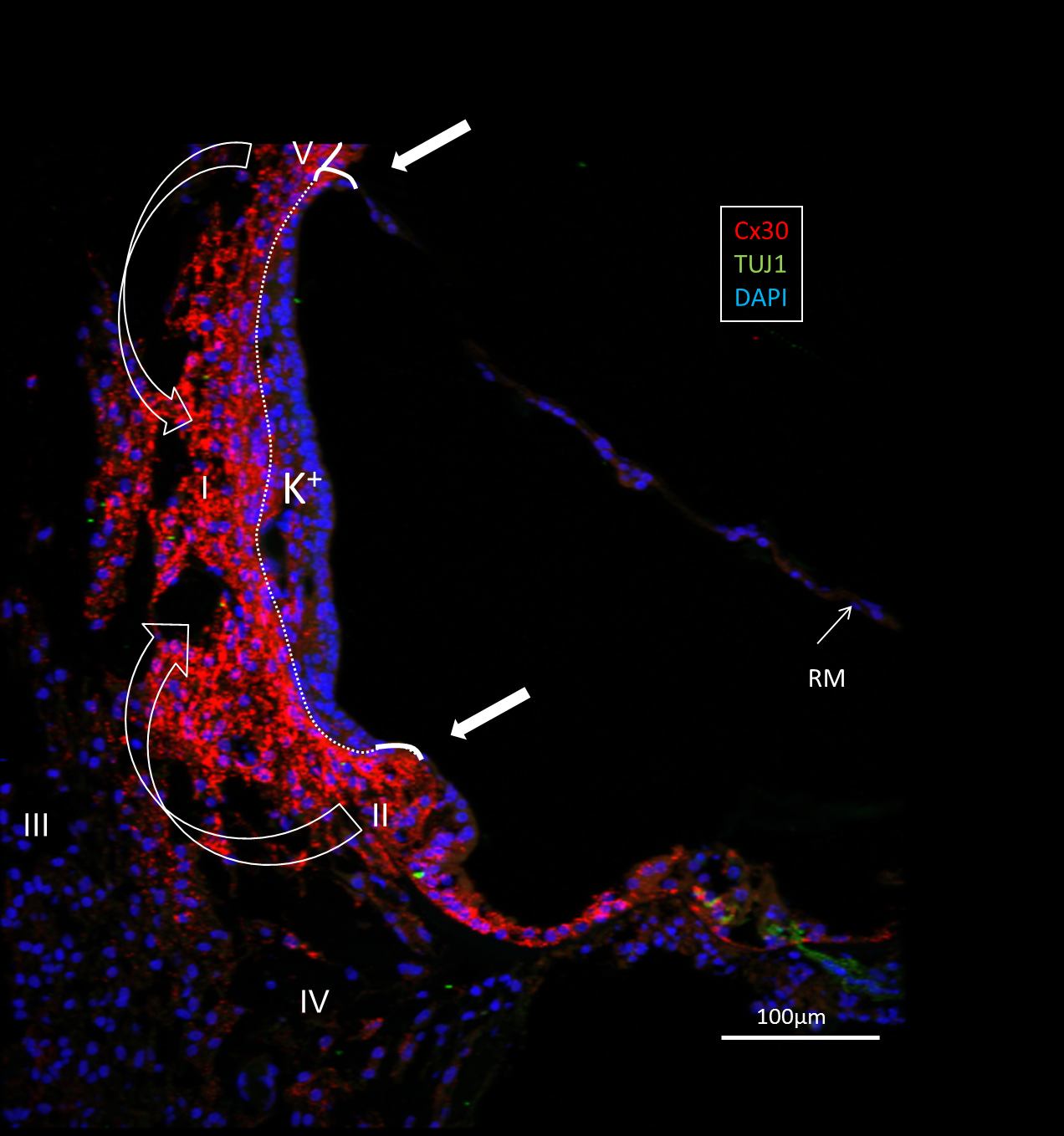

Supplement: FIGURE S1 — Cx30 expressing GJs form a connecting syncytium between subpopulations of fibrocytes (I, II and V) to the StV. Epithelial Cx30 expression is also seen in supporting cells of the organ of Corti, Claudius cells and outer sulcus cells. The Claudin-11 barrier is outlined (dotted line) with its anchoring sites (bold arrows). [file Image_1.TIFF]

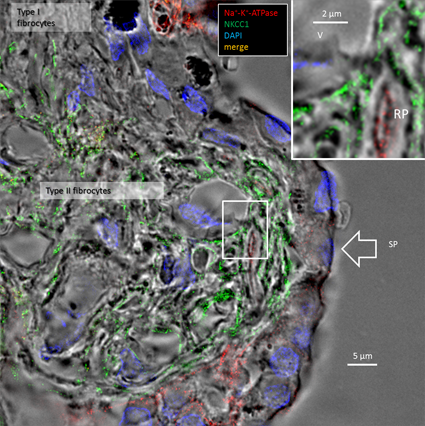

Supplement: FIGURE S2 — Combined bright-field and SR-SIM shows Na/K/Cl cotransporter expressed around the SP blood vessels. The same region shown as in Figure 8B (the inset shows the boxed area at a higher magnification). The root cell process (RP) expresses Na/K-ATPase (α1-subunit). SP, spiral prominence; V, vessels in the spiral prominence. [file Image_2.TIFF]

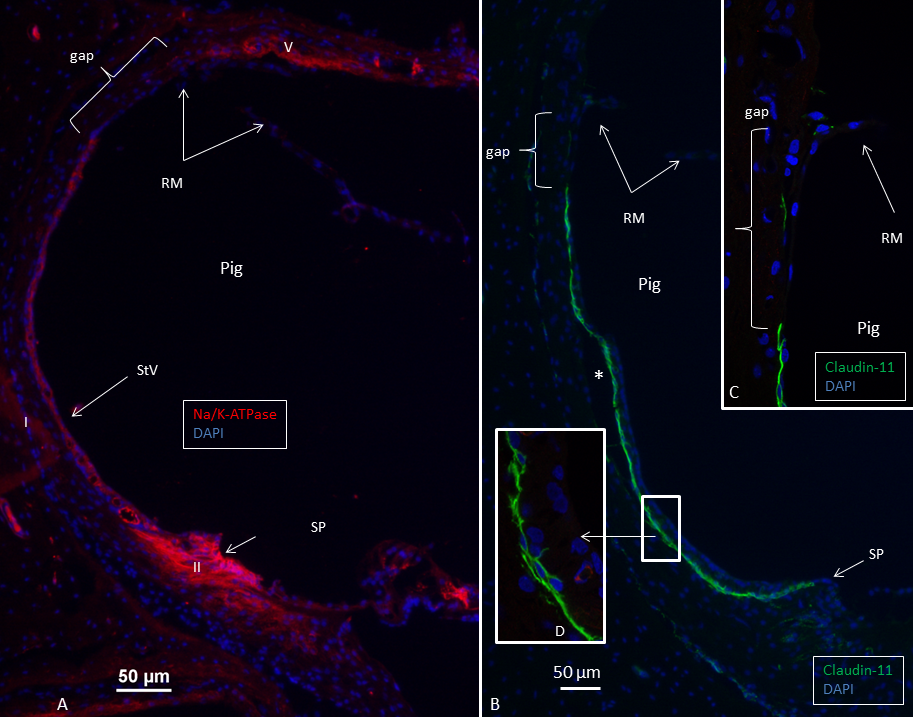

Supplement: FIGURE S3 — Na/K-ATPase (β1-subunit) and Claudin-11 expression in the pig cochlea. (A) Na/K-ATPase is heavily expressed in the type II and V fibrocytes and in the stria vascularis marginal cells. There is also a strong expression in the organ of Corti and spiral limbus (displaced tympanic covering layer). (B) Claudin is expressed in a thin band reaching from the spiral prominence to the region near the Reissner’s membrane. There is no Claudin-11 expression in the suprastrial region (∗; StV epithelium slightly separated). (C) Confocal microscopy. There is no Claudin expression at a region (gap) beneath the insertion point of RM (arrow). SP, spiral prominence; StV, stria vascularis. Type I, II and V fibrocytes. RM, Reissner’s membrane. [file Image_3.TIF]
